# The relationship between stress shielding, bone density changes and implant migration, failure and fracture after total knee arthroplasty: A systematic review

**DOI:** 10.1002/jeo2.70350

**Published:** 2025-07-13

**Authors:** Giulio Senesi, Giuseppe Barone, Salvatore Pinelli, Maria Scoppolini Massini, Raffaele Zinno, Domenico Alesi, Erika Pinelli, Laura Bragonzoni

**Affiliations:** ^1^ Department for Life Quality Studies Alma Mater Studiorum University of Bologna Rimini Italy; ^2^ IRCCS, Istituto Ortopedico Rizzoli, 2nd Orthopaedic and Traumatologic Clinic Bologna Italy

**Keywords:** bone mineral density, implant loosening, implant migration, stress shielding, total knee arthroplasty

## Abstract

**Purpose:**

Total knee arthroplasty (TKA) represent a well‐established treatment for advanced osteoarthritis. Despite advancements in implant design and materials, the impact of stress shielding on bone mineral density (BMD) and its relationship with implant migration, loosening, and fracture remains insufficiently explored. This study aims to evaluate the association between stress shielding, BMD changes, and implant outcomes after TKA.

**Methods:**

A systematic review was performed following the Preferred Reporting Items for Systematic Reviews and Meta‐Analysis (PRISMA) guidelines. Four major databases (MEDLINE (PubMed), Cochrane Central Register of Controlled Trials (CENTRAL), Web of Science (WOS), EMBASE) were thoroughly reviewed.

**Results:**

A total of 491 studies were initially identified, of which nine met the inclusion criteria and were included in the final review. Eight were randomised controlled trials, and one was a prospective observational study. All studies were conducted in clinical settings, comprising a total of 467 patients. BMD variations due to stress shielding were most pronounced during the first 6–12 months postoperatively. However, except for one isolated case, no direct correlation was identified between BMD changes caused by stress shielding and implant migration, loosening or fracture.

**Conclusions:**

This systematic review highlights that variations in bone mineral density due to stress shielding are not directly correlated with implant failure, migration or loosening in TKA. Stress shielding remains a critical phenomenon, particularly in the early postoperative phases, emphasising the importance of optimised materials, fixation methods and implant designs to reduce bone loss and improve long‐term stability. Due to its recognised occurrence in clinical practice, further in vivo studies would contribute to a better understanding of the stress shielding.

**Level of Evidence:**

Level II, systematic review of studies.

**Abbreviations**
BIC: bone‐implant contact ratio
BMD: bone mineral density
BSAP: bone‐specific alkaline phosphatase
CTx: collagen type 1 crosslinked C‐telopeptide
DEXA: dual‐energy x‐ray absorptiometry
FS HA: Freeman–Samuelson Hydroxyapatite
HSS: Hospital for Special Surgery
JBI: Joanna Briggs Institute
KOOS: Knee Injury and Osteoarthritis Outcome Score
KSS: Knee Society Score
MG II: Miller–Galante II
OKS: Oxford Knee Score
P1NP: plasmaosteocalcin serum type 1 procollagen *N*‐terminal
PEEK: polyether‐ether‐ketone
PICOS: Patients, Interventions, Comparators, Outcomes, and Study design
PRISMA: Preferred Reporting Items for Systematic Reviews and Meta‐analyses
RCTs: randomised controlled trials
RLL: radiolucent lines
ROI: regions of interest
RSA: radiostereometric analysis
TKA: total knee arthroplasty
WOS: Web of Science

## INTRODUCTION

Total knee arthroplasty (TKA) remains a solid procedure to relieve pain and restore mobility in patients with advanced osteoarthritis [8]. Despite the advancements in design, materials and surgical technique, revision rates remain a significant concern, with approximately 6% of cases requiring revision within five years and 12% within ten years [22]. Implant loosening, the leading cause of TKA failure (39.9%), is mainly due to factors such as implant wear, infection [5] and stress shielding [11]. Stress shielding, a biomechanical phenomenon, arises from the significant stiffness mismatch between implants and bone. This imbalance reduces the mechanical stimulus necessary for bone maintenance, often leading to remodelling, decreased bone mineral density (BMD) [2, 24, 36, 45, 63] and subsequent periprosthetic fractures and increased implant instability [62]. Research indicates that BMD loss is particularly pronounced during the first 6–12 months post‐surgery, with reductions of up to 40% observed in areas adjacent to tibial and femoral components [17, 41]. In TKA this early bone loss may compromise implant longevity, potentially resulting in aseptic loosening, implant migration, and eventual failure or fracture [14, 34, 46]. One of the indicators of implant loosening is represented by early migration of the tibial component [48, 52]. Thus, it serves as a reliable indicator of long‐term prosthetic survival [47]. In addition, bone density loss, when accompanied by micromotion, substantially heightens the risk of implant loosening and failure [31, 55]. Between the radiographic techniques, Radiostereometric Analysis (RSA) has been considered suitable for recognising implant micromotions before they become clinically evident [19]. Another parameter which shows the integration between implant and bone is the variation of BMD in the periprosthetic bone [26, 49]. Several imaging techniques have been adopted to monitor this parameter, mostly based on X‐ray [27].

To date, no systematic review has specifically examined the relationship between implant migration, loosening and fracture in correlation with BMD changes and their potential link to stress shielding. This review aims to systematically evaluate the possible correlation between these factors and the role of stress shielding.

## METHODS

The systematic review method was conducted in accordance with the Preferred Reporting Items for Systematic Reviews and Meta‐analyses (PRISMA) guidelines [37]. Literature research was considered in line with ethical principles for medical research involving human subjects. No approval was required for this review.

### Inclusion and exclusion criteria

The following Patients, Interventions, Comparators, Outcomes, and Study design (PICOS) framework was used to develop the search strategy: (P) Patients undergoing primary TKA; (I) TKA and BMD analysis; (C) No comparator or other surgical techniques/prosthetic designs; (O) Prosthesis failure (loosening, implant breakage), migration, fracture and possible associated factors, if present (e.g., stress shielding); (S) Randomised controlled trials (RCTs) and Observational studies. The inclusion criteria were the following: (i) language: articles written in English; (ii) population of interest: patients undergoing primary TKA; (iii) intervention: BMD analysis and TKA; (iv) outcome: at least one prosthesis failure, migration, or fracture, and possible associated factors, if present (e.g., stress shielding); (v) paper: full text available. The exclusion criteria were as follows: (i) study design: review articles; (ii) language: articles not written in English; (iii) intervention: studies without BMD analysis or TKA; (iv) outcome: studies without reported prosthesis failure, migration, or fracture.

### Search strategy and selection process

The search strategies were developed starting with the creation of a search string composed of specific terms combined with Boolean operators. This search query was subsequently adapted and applied to the following databases: MEDLINE (PubMed), Cochrane Central Register of Controlled Trials (CENTRAL), Web of Science (WOS), and EMBASE. The keywords used to construct the search query were: ‘Total Knee Replacement/Arthroplasty/Prosthesis’, ‘Bone Mineral Density’, ‘Stress Shielding/Prosthesis Migration/Loosening/Fracture’. The complete search query used on MEDLINE (PubMed) is available in Supporting Information (Annex A – Search Strategy). Additionally, a grey literature search was conducted using manual researches of key conference proceedings, journals, professional organisation websites, and guideline exchange centres. Again, the snowball technique was used to explore references cited in the primary articles, identifying potential studies that met the eligibility criteria and could be included in this review. From the full list of articles found for each database, duplicate articles were excluded using EndNote (EndNote X9.3.3) and then manually. Based on the PICOS criteria, the title and abstract were initially assessed by two authors (GS and GB). The full texts of these potentially eligible studies were reviewed for inclusion by the same six authors (GS, GB, SP, MSM, RZ and EP), who independently assessed each study. In addition, a snowball technique was applied by checking the reference lists of the included articles to identify further relevant studies that might have been missed in the database search. Data extraction from included studies was performed by six authors (GS, GB, SP, MSM, RZ and EP).

### Quality assessment and risk of bias

A risk of bias critical appraisal of each article included in the review was conducted independently and blinded by two authors (GS and GB), using the Joanna Briggs Institute (JBI) Critical Appraisal Checklists for randomised control trial [1] and the JBI Critical Appraisal Checklists for Cohort Studies [38]. Each item was scored 'yes', 'no', 'unclear' or 'not applicable'. All the authors decided and approved the criteria to generate the overall score as recommended by the JBI manual. In detail, the overall score was assigned as follows: (i) 'high quality' if all the criteria were met; (ii) 'medium quality' if one or more domains were unclear; (iii) 'low quality' if one or more domains were not met.

## RESULTS

### Study selection

**Figure 1:**
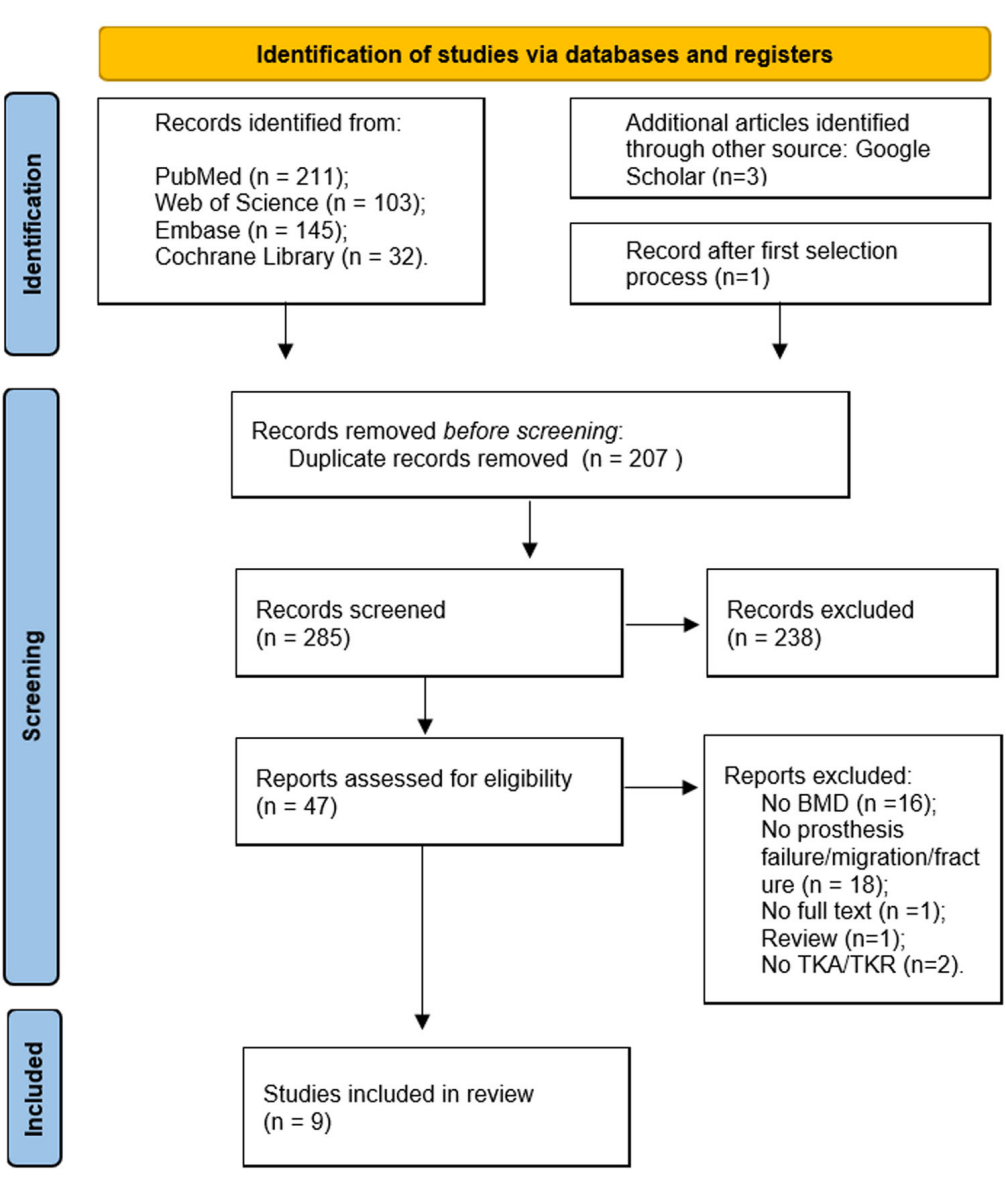
PRISMA flow diagram of the selection of studies.

The search in the four databases produced a total of 491 articles. After removing 207 duplicates, 284 articles remained. These underwent title and abstract screening, resulting in 46 eligible articles that were subjected to full‐text screening. A total of 38 articles were excluded as they did not meet the necessary inclusion criteria. In addition, using the snowball technique, three more articles were identified, and one of them was included. Finally, nine articles [7, 29, 30, 39, 42, 51, 56, 57, 59] were included in the study. In Figure 1, the PRISMA flowchart [43] shows the search strategy process.

### Studies design and sample characteristics

The included studies consisted of eight RCTs [7, 30, 39, 42, 51, 56, 57, 59] and one observational prospective study [29]. All studies have been conducted in clinical settings. The total sample size included in this systematic review consisted of 467 patients, with data aggregated from all the studies analysed [7, 29, 30, 39, 42, 51, 56, 57, 59]. The overall mean age of the patients was 66.6 years. The overall mean BMI was 29.3 kg/m². In Supporting Information (Annex A – Table S1) the data extraction of all sample characteristics are available, as reported in the included studies.

### Implant design

Based on the fixation methods, we classified the implants as 'Cemented' and 'Cementless' (subdivided in 'Porous‐coated' and 'Porous‐coated with additional materials'). Details are shown in Table 1.

**Table 1:** Summary of prostheses classification.

Author	Prosthesis name	Implant material	Implant bone interface	Interface classification
Règner et al. [51]	Uncemented freeman‐samuelson hydroxyapatite (FS HA) or Miller–Galante II (MG II) total knee arthroplasty.	FS HA = Not specified MG = Titanium	FS HA = hydroxyapatite (HA) coating MG II = porous‐coated surface	CPC HA; CPC
Tägil et al. [57]	Porous‐coated anatomic (PCA Modular; Howmedica, Rutherford, NJ)	Co–CR–Mo alloy	Porous‐coated surface; high‐viscosity cement; low‐viscosity cement	CPC; C
Nivbrant et al. [42]	Advanced Coated System (ACS) Fixed‐Bearing Posterior Stabilised total knee replacement components (Implantcast)	Co–Cr–Mo alloy with porous coating, titanium nitride (TiN) ceramic layer, and UHMW polyethylene inserts	Porous coating with a 5.5‐mm titanium nitride (TiN) ceramic layer	CPC TiN; C
Linde et al. [30]	Regenerex (Zimmer Biomet, Warsaw, IN, USA)	Titanium	Porous titanium	CPC; C
Dyreborg et al. [7]	Vanguard total knee system (Zimmer Biomet, Warsaw, IN, USA)	VP = cobalt‐chromium tibial implants VR = titanium	Porous plasma spray (VP) and porous titanium (Regenerex ‐ VR)	CPC plasma; CPC
Tjørnild et al. [59]	P.F.C. (Press‐Fit Condylar) Sigma Cruciate Retaining TKA (DePuy International, Leeds, UK)	Co–Cr alloy tibial components, UHMWPE	Cemented Simplex bone and cement (Stryker)	C
Li and Nilsson [27]	Miller‐Galante II (Zimmer, Warsaw, IN, USA).	Titanium	Porous‐coated surface and cement	CPC; C
Stilling et al. [56]	Maxim Total Knee (Zimmer Biomet, Warsaw, IN, USA)	Co–Cr components, gamma‐sterilised UHMW polyethylene inserts	Palacos R bone cement	C
Mosegaard et al. [39]	Vanguard prosthesis (Zimmer Biomet, Warsaw, IN, USA)	Co–Cr tibial tray and cruciate‐retaining femoral component, ArCom UHMW polyethylene 3‐peg patella button, modular polyethylene liner	Refobacin Plus or standard Refobacin bone cement	C

Abbreviations: C, cemented; CPC, cementless porous‐coated.

### Implant migration, bone density changes and fixation methods

Eight studies showed no correlation between implant migration, BMD changes and fixation methods [7, 29, 30, 39, 42, 51, 56, 57, 59]. All the data related are reported in Table 2.

**Table 2:** Summary of correlation between migration and BMD.

Author	Migration analysis	BMD analysis	Migration results	BMD results	Fixation methods
Tjørnild et al. [59]	RSA	DXA	MTPM a 24 month: Fixed Bearing (FB) = 0.69 mm; Mobile Bearing (MB) = 0.55 mm; no diff. (*p* = 0.1); Migration stable after 3 months; Total Translation = > FB; Total Rotation = similar	Similar reduction between FB and MB	C
Linde et al. [30]	RSA	DXA	Cemented MTPM = 0.73 mm (12 months); stable 12‐24 months; MTPM = 0.02 mm	Similar reduction in ROI −1 and ROI −2	C
			MTPM = 1.63 mm at 12 months; stabilised afterward	Temporary increase in ROI −1 and ROI −2, followed by reduction similar to cemented implants	CPC
Li and Nilsson [27]	RSA	DEXA	40% migration in 3 months; stability at 24 months	Initial reduction; recovery to baseline within 24 months	C
			70% of total migration occurred within 3 months; gradual decrease thereafter	12.5% reduction at 3 months, return to baseline by 24 months; high variability	CPC
Stilling et al. [56]	RSA	DXA	21 tibial components (MTPM > 0.2 mm) between 1 and 2 years; at 2 years 5 IS > 0.7° rotation y axis. Finned Stem >0.7°	Greater reduction in I‐beam (IS) group (Medial: −13%, Posterior: −13%); bone loss below the steam was higher in IS in the first year = −6%	C
Mosegaard et al. [39]	RSA	DXA	Refobacin Plus (RP): Rotation around y‐axis +0.21° vs. Refobacin Bone Cement (RR) (6–12 months, not significant); Total Traslation: RR > RP ( + 0.23 mm at 12 months, +0.25 mm at 24 months, *p* = 0.01); MTPM at 2 years: (RR) + 0.32 mm (*p* = 0.04).	Similar reduction between RR and RP groups	C
Nivbrant et al. [42]	RSA	DXA	Maximum subsidence: 0.68 mm (12 months); no further subsidence	Medial: −12.7%; Lateral: −7.4%; Anterior: −12.2%	C
			Higher migration vs cemented; subsidence >1 mm in 12/29 implants at 12 months; Mean rotation into flexion = 0.59° at 3 months	Less medial BMD loss vs cemented; significant differences in medial, lateral, and anterior regions	CLM
Tägil et al. [57]	RSA	Tomography bone mineral density (CTBMD)	MTPM = 1.0 mm at 1 year, uniform trend	Consistent total volumetric bone and osteoid surface	C
			Uniform migration trends observed	Higher trabecular bone in rheumatic knees medially and laterally compared to osteoarthritic knees	CPC
Regnér et al. [51]	RSA	TXA	Higher instability compared to uncemented freeman‐samuelson hydroxyapatite, subsidence significantly higher	15% reduction at 1 year, stabilised thereafter	CPC
			Minimal migration after 1 year; significantly less subsidence vs MG II implants (*p* = 0.01)	29% reduction at 1 year, 36% at 4–5 years; 72% showed trabecular bone condensation near the stem	CLM
Dyreborg et al. [7]	RSA	DEXA	Stabilisation after 24 months, MTPM = 1.8 mm at 5 years	Minor reductions or stabilisation; ROI −1: −5.9%, ROI −2: −4.6%, ROI −3: +3.1%	CPC
			Stable migration patterns after 24 months; MTPM = 1.4 mm at 5 years. Between 24‐60 months MTPM = 0.3	Minor reductions or stabilisation; ROI −1: −5.0%, ROI −2: −4.6%, ROI −3: +2.1%. BMD at 60 months was 1.053 g/cm^2^	CLM

Abbreviations: C, cemented; CLM, cementless with additional materials; CPC, cementless; DEXA, dual‐energy x‐ray absorptiometry; MTPM, maximal total point motion; ROI, region of interest; RSA, radiostereometric analysis; TXA, total knee arthroplasty.

In addition, no stress shielding was observed in these studies [7, 29, 30, 39, 42, 51, 56, 57, 59]. Only Stilling et al. [56] demonstrated a significant association between medial BMD loss and continuous migration (rho –0.38; *p* = 0.03). This finding is in line with the greater stress shielding observed in the I‐beam block stem (IS) group, which showed significantly higher bone loss compared to the finned stem (FS) group. Specifically, stress shielding was more pronounced in the medial and posterior regions around the IS (–13% vs. –2%, *p* < 0.01) and below the stem (–6% vs. +1%, *p* = 0.01) during the first two years. Between 2 and 5 years, bone loss decreased markedly for FS (*p* < 0.002).

### Implant loosening/fracture, bone density changes and fixation methods

The included studies reported various complications. One intraoperative tibial plateau fracture occurred in the cemented group [42] requiring screw fixation. One periprosthetic fracture was reported in the mobile‐bearing group [59]. No instances of implant breakage were identified across the studies. Two cases of revision due to pain were highlighted. In one case, a cementless tibial component was revised at 12 months due to persistent pain, despite minimal subsidence of 0.25 mm. Poor bone ingrowth at the porous interface and moderate detritic synovitis were observed upon retrieval analysis [42]. In another case, significant migration (maximal total point motion 1.6 mm) at one year necessitated revision 3 years postoperatively, where the tibial component was found to be loose and easily removable [57]. Aseptic loosening was reported in one case. The revision revealed florid synovitis, extensive patellar component wear, and loosening of the tibial component [57]. Infections were reported in four patients [7, 42, 59]. Revisions due to malalignment were less frequent, with one case involving tibial and femoral components revised due to femoral malalignment in the cementless group [42]. Additionally, one patient in the fixed‐bearing group and another in the mobile‐bearing group required reoperations however, the specific reasons for these reoperations were not provided in the study [59]. Finally, knee instability accounted for two revisions, both described by Dyreborg et al. [7]. None of these cases showed any link to BMD changes and stress shielding.

### Other results

The studies investigated various aspects of patient‐reported outcomes, biomarkers, radiolucent lines (RLL), and bone cement thickness. The Hospital for Special Surgery (HSS) score demonstrated excellent results in both the Freeman–Samuelson Hydroxyapatite (FS HA) and Miller–Galante II (MG II) total knee arthroplasty groups at 5 years [51]. Improvements were observed in the Oxford Knee Score (OKS) [39], the Knee Society Score (KSS) [7] and the American Knee Society Score (AKSS) [56]. Further gains were documented in the Knee Injury and Osteoarthritis Outcome Score (KOOS), particularly in the physical functioning and pain subscales, as well as in the SF‐36 [39]. Biomarkers of bone turnover provided insight into the postoperative bone remodelling process. Linde et al. [30] reported significantly elevated serum collagen type 1 crosslinked C‐telopeptide (CTx) levels in the cementless group during the first 2 weeks postoperatively (*p* = 0.02). Other biomarkers, including plasmaosteocalcin serum type 1 procollagen *N*‐terminal (P1NP), osteocalcin, and serum bone‐specific alkaline phosphatase (BSAP), normalised by 6 months, reflecting a return to baseline bone remodelling activity. Radiolucent lines were more commonly observed in cementless implants (14 of 17 patients) compared to cemented ones (3 of 17) at 6 months (*p* < 0.001) [30]. However, the prevalence of RLL decreased significantly by two years, suggesting stabilisation of the implants over time. Variations in bone cement thickness were analysed, revealing that medial cement thickness was significantly greater in the Refobacin bone cement (RR) group compared to the Refobacin Plus cement (RP) group (2.35 mm vs. 2.00 mm, *p* < 0.01), while lateral thickness remained similar [39]. Additionally, RR was rated as more user‐friendly by surgeons compared to RP cement (*p* < 0.01).

### Risk of bias

Table 3 summarises the quality assessment and risk of bias for each study, evaluated using the JBI tool [1, 38]. Detailed results are available in Supporting Information (Annex A – Table S2).

**Table 3:** General score of quality assessment and risk of bias.

Authors	Study design	Tool for assessment	Quality
Règner et al. [51]	RCT	JBI for RCT [1]	Low
Tägil et al. [57]	RCT	JBI for RCT [1]	Low
Nivbrant et al. [42]	RCT	JBI for RCT [1]	High
Li and Nilsson [27]	RCT	JBI for RCT [1]	High
Dyreborg et al. [7]	RCT	JBI for RCT [1]	Low
Tjørnild et al. [59]	RCT	JBI for RCT [1]	High
Stilling et al. [56]	RCT	JBI for RCT [1]	Low
Mosegaard et al. [39]	RCT	JBI for RCT [1]	Low
Li and Nilsson [28]	Observational prospective study	JBI for Cohort Studies [38]	Low

Abbreviations: JBI, Joanna Briggs Institute; RCT, randomised controlled trial.

## DISCUSSION

The main findings of this systematic review suggest that bone density variations, while potentially influenced by stress shielding, show no direct correlation with implant migration, failure or loosening. Only one study, conducted by Stilling et al. [56], demonstrated a correlation between these factors (rho –0.38; *p* = 0.03). This study identified significant bone loss specifically in the medial and posterior regions (–13% vs. –2%, *p* < 0.01) and below the stem (–6% vs. +1%, *p* = 0.01) during the first two years in the I‐beam block stem group. The cobalt‐chromium composition of the cemented prosthesis used in this study provides high durability, but its stiffness exceeds the one of the bone, contributing to localised bone resorption and instability [35]. These findings underline the importance of material properties in influencing periprosthetic bone remodelling and implant stability, suggesting that materials with a lower Young's modulus (e.g., titanium or PEEK) better mimic bone properties and elasticity, promoting a balanced remodelling [10, 12, 21, 32, 35, 54, 61]. The articles included in this review do not reveal a direct correlation due to stress shielding. This can be attributed to the multifactorial nature of this process, influenced by factors such as implant surface roughness [50], fixation methods [3], bone quality [33] and implant alignment [13, 25]. Implant surface roughness, in particular, enhances the bone‐implant contact ratio (BIC), optimises force distribution and minimises the risk of micromovements [16]. Hériveaux et al. [16], through a finite element analysis, found that a heterogeneous distribution of maximum shear stress was evident in the periprosthetic bone tissue, with high interface stress in regions with low implant roughness and underloaded areas near the roughness valleys. These findings underscore the importance of optimising surface roughness and maximising the BIC ratio to improve osseointegration, reduce micromotion, and achieve a more uniform stress distribution. Furthermore, maximising the BIC ratio not only enhances osseointegration phenomena but also minimises stress‐shielding effects, which predominantly occur in bone tissue regions corresponding to roughness valleys [50]. Micromotion, defined as an excessive movement at the bone‐implant interface, can disrupt osseointegration and lead to progressive bone loss over time [20]. Fixation methods represent another aspect in minimising implant migration and promoting long stability. Cemented fixation provides excellent primary stability [44], however, long‐term mechanical separation at the bone‐cement interface can lead to localised bone resorption and instability [6, 15]. Cementless fixation, on the other hand, relies on biological bone internal growth, which reduces micromotion and shear stress at the interface, achieving superior long‐term stability despite challenges in early osseointegration [6]. Many of the studies included in this review highlighted an early stabilisation of implant migration, typically observed within the first 12–24 months postoperatively. For cemented implants, migration patterns showed stabilisation as early as 12 months, with minimal or no changes observed between 12 and 24 months [30, 39]. For cementless implants, stabilisation has occurred in a similar period of time, although there is some variability in the initial subsidence or micromotion [7, 42]. Bone quality not only affects implant stability but also influences the choice of prosthesis [53]. Cemented implants are often preferred in cases of poor bone quality, while cementless designs are better suited for patients with sufficient bone density to support osseointegration [40]. The alignment of the implant is essential for achieving optimal load distribution, preventing stress concentration, and reducing the risk of loosening and failure. Varus or valgus misalignment, particularly post‐operative, increases eccentric load distribution, creating fulcrum points that amplify edge loading and stress concentrations, contributing to implant loosening and failure [13, 25]. Edge loading occurs when forces are unevenly distributed across the tibial plateau, concentrating stress at the margins of the implant [23]. This localised stress can accelerate wear of the prosthetic materials, exacerbate bone resorption, and compromise the overall stability of the implant. Tailored surgical strategies, including precise alignment during implantation and the choice of fixation method and implant material, are essential to address these individual variations and minimise the risk of edge loading [60]. Considering the multifactorial nature of stress shielding, its effects can be mitigated, and implant stability enhanced through targeted actions such as: preoperative screening of high‐risk patients using DEXA [18, 28], accurate preoperative planning [58] and selecting the most appropriate implant type based on patient‐specific factors [9]. Despite these considerations, variations in BMD remain an important factor in providing insights into the interplay between stress shielding, implant migration and long‐term stability, although no direct association with failure, loosening, or migration has been identified in this review. Borsinger et al. [4] analysed the distribution of BMD in the proximal tibia and its implications for TKA. The results show a progressive decrease in BMD from proximal to distal regions, with the most significant variations occurring within ±2 mm of the tibial cut, a critical area for implant stability. Reductions in BMD in this review were observed mainly during the first 6–12 months postoperatively [29, 51, 56]. This initial loss was followed by stabilisation or partial recovery in some cases, as highlighted by Li et al. [29], where a 12.5% reduction at three months returned to baseline within 24 months. Cemented implants [30, 39, 56, 59] generally exhibited more stable BMD trends, with comparable reductions across regions of interest (ROI). In contrast, the cementless implants, especially porous coated ones, have shown different results. Some studies in fact report a median loss of BMD less pronounced than cemented counterparts [29, 42, 51].

Two implications emerge from this review. Implant materials and fixation methods play a critical role not only in ensuring implant stability but also in mitigating the effects of stress shielding, which can lead to localised bone resorption. Finally, early BMD loss underscores the importance of developing strategies to prevent excessive bone resorption and maintain periprosthetic bone health.

In the end, the articles included in this systematic review did not identify a correlation between changes in bone density and migration, loosening or implant fracture due to stress shielding. However, the presence of stress shielding is well documented in clinical practice [3, 6, 13, 21, 25, 33, 35]. Therefore, further in vivo studies are essential to enhance the knowledge of this phenomenon.

This review is subject to several limitations. In particular, there is marked heterogeneity in study designs, follow‐up durations, patient demographics, implant designs, and bone–prosthesis interfaces. Notably, although all studies except one are RCTs, none share a common control group, and the variability in prosthesis types and osseointegration methods—as detailed in Annex 1—precludes the possibility of performing a uniform pooled quantitative analysis. These intrinsic differences limit the generalisability and statistical robustness of our combined results. Future research should prioritise large‐scale, prospective studies with uniform methodologies to clarify the interactions between BMD changes, implant migration, loosening and fracture. Understanding the interplay between implant material, design, fixation techniques and patient‐specific factors is essential for optimising outcomes in total knee arthroplasty.

## CONCLUSION

In conclusion, this systematic review highlights that variations in bone mineral density due to stress shielding are not directly correlated with implant failure, migration or loosening in TKA. Stress shielding remains a critical phenomenon, particularly in the early postoperative phases, emphasising the importance of optimised materials, fixation methods and implant designs to reduce bone loss and improve long‐term stability. Due to its recognised occurrence in clinical practice, further in vivo studies would contribute to a better understanding of the stress shielding.

## AUTHOR CONTRIBUTIONS

*Conceptualisation*: Giulio Senesi and Laura Bragonzoni. *Methodology*: Giulio Senesi, Salvatore Pinelli and Giuseppe Barone. *Supervision*: Raffaele Zinno, Maria Scoppolini Massini and Laura Bragonzoni. Writing—original draft, Giulio Senesi, Salvatore Pinelli and Giuseppe Barone. *Writing—review and editing*: Domenico Alesi, Erika Pinelli and Laura Bragonzoni. All authors have read and agreed to the published version of the manuscript.

## CONFLICT OF INTEREST STATEMENT

The authors declare no conflicts of interest.

## ETHICS STATEMENT

None declared.

## Supporting information

Supporting information.

Supporting information.

Supporting information.

## Data Availability

The data that supports the findings of this study are available in the supplementary material of this article.
