# Supplementary material for: The relationship between stress shielding, bone density changes and implant migration, failure and fracture after total knee arthroplasty: A systematic review
Source: J Exp Orthop. 2025 Jul 13;12(3):e70350. doi: 10.1002/jeo2.70350 (PMC12255957; doi:10.1002/jeo2.70350)
Supplement: Supplementary file 1 — Supporting information. [file JEO2-12-e70350-s001.docx]

((TKA[Title/Abstract]) OR (TKR[Title/Abstract]) OR (Total Knee Replacement[Title/Abstract]) OR (Total Knee Arthroplasty[Title/Abstract]) OR (Total knee prosthesis[Title/Abstract]))

AND

((Bone Remodeling[Title/Abstract]) OR (Bone Loss[Title/Abstract]) OR (Osteopenia[Title/Abstract]) OR (Osteoporosis[Title/Abstract]) OR (Bone Mass[Title/Abstract]) OR (Bone Turnover[Title/Abstract]) OR (Bone Metabolism[Title/Abstract]) OR (Bone Density[Title/Abstract]) OR (Bone Losses[Title/Abstract]) OR (Bone Densities[Title/Abstract]) OR (Bone Mineral Density[Title/Abstract]) OR (Bone Mineral Densities[Title/Abstract]) OR (Bone Mineral Content[Title/Abstract]) OR (Bone Mineral Contents[Title/Abstract]) OR (Bone Resorption[Title/Abstract]))

AND

((Stress Shielding[Title/Abstract]) OR (Mechanical Loading [Title/Abstract]) OR (Implant Stiffness[Title/Abstract]) OR (Bone-Implant Interaction[Title/Abstract]) OR (Bone Stress Reduction[Title/Abstract]) OR (Mechanical Stress[Title/Abstract]) OR (Mechanical Stresses[Title/Abstract]) OR (Load Transfer[Title/Abstract]) OR (Mechanical Shielding[Title/Abstract]) OR (Stress Redistribution[Title/Abstract]) OR (Load Sharing[Title/Abstract]) OR (Stress Protection[Title/Abstract]) OR (Fracture, Knee[Title/Abstract]) OR (Knee Fracture[Title/Abstract]) OR (Knee Joint Fractures[Title/Abstract]) OR (Fracture, Knee Joint[Title/Abstract]) OR (Joint Fracture, Knee[Title/Abstract]) OR (Knee Joint Fracture[Title/Abstract]) OR (Femoral Condyle Fractures[Title/Abstract]) OR (Condyle Fracture, Femoral[Title/Abstract]) OR (Condyle Fractures, Femoral[Title/Abstract]) OR (Femoral Condyle Fracture[Title/Abstract]) OR (Fracture, Femoral Condyle[Title/Abstract]) OR (Femoral Condyles Fractures[Title/Abstract]) OR (Condyles Fracture, Femoral[Title/Abstract]) OR (Femoral Condyles Fracture[Title/Abstract]) OR (Fracture, Femoral Condyles[Title/Abstract]) OR (Tibial Eminence Fractures[Title/Abstract]) OR (Eminence Fracture, Tibial[Title/Abstract]) OR (Fractures, Tibial Eminence[Title/Abstract]) OR (Fracture, Tibial Eminence[Title/Abstract]) OR (Tibial Eminence Fracture[Title/Abstract]) OR (Tibial Tuberosity Fractures[Title/Abstract]) OR (Fracture, Tibial Tuberosity[Title/Abstract]) OR (Tibial Tuberosity Fracture[Title/Abstract]) OR (Tuberosity Fractures, Tibial[Title/Abstract]) OR (Tuberosity Fracture, Tibial[Title/Abstract]) OR (Tibial Spine Fractures[Title/Abstract]) OR (Fracture, Tibial Spine[Title/Abstract]) OR (Spine Fracture, Tibial[Title/Abstract]) OR (Tibial Spine Fracture[Title/Abstract]) OR (Fractured Knee[Title/Abstract]) OR (Fractured Knees[Title/Abstract]) OR (Knee, Fractured[Title/Abstract]) OR (Knees, Fractured[Title/Abstract]) OR (Fracture, Stress[Title/Abstract]) OR (Stress Fracture[Title/Abstract]) OR (Fatigue Fractures[Title/Abstract]) OR (Fatigue Fracture[Title/Abstract]) OR (Fracture, Fatigue[Title/Abstract]) OR (Fractures, Fatigue[Title/Abstract]) OR (Fractures, March[Title/Abstract]) OR (Fracture, March[Title/Abstract]) OR (March Fracture[Title/Abstract]) OR (March Fractures[Title/Abstract]) OR (Stress Fractures[Title/Abstract]) OR (Insufficiency Fractures[Title/Abstract]) OR (Fracture, Insufficiency[Title/Abstract]) OR (Insufficiency Fracture[Title/Abstract]) OR (Microfractures[Title/Abstract]) OR (Microfracture[Title/Abstract]) OR (Micro Fractures[Title/Abstract]) OR (Fracture, Micro[Title/Abstract]) OR (Fractures, Micro[Title/Abstract]) OR (Micro Fracture[Title/Abstract]) OR (Bone Stress Reaction[Title/Abstract]) OR (Bone Stress Reactions[Title/Abstract]) OR (Stress Reaction, Bone[Title/Abstract]) OR (Failure, Prosthesis[Title/Abstract]) OR (Failures, Prosthesis[Title/Abstract]) OR (Prosthesis Failures[Title/Abstract]) OR (Prosthesis Loosening[Title/Abstract]) OR (Loosening, Prosthesis[Title/Abstract]) OR (Loosenings, Prosthesis[Title/Abstract]) OR (Prosthesis Loosenings[Title/Abstract]) OR (Prosthesis Migration[Title/Abstract]) OR (Migration, Prosthesis[Title/Abstract]) OR (Migrations, Prosthesis[Title/Abstract]) OR (Prosthesis Migrations[Title/Abstract]) OR (Prosthesis Durability[Title/Abstract]) OR (Durabilities, Prosthesis[Title/Abstract]) OR (Durability, Prosthesis[Title/Abstract]) OR (Prosthesis Durabilities[Title/Abstract]) OR (Prosthesis Survival[Title/Abstract]) OR (Prosthesis Survivals[Title/Abstract]) OR (Survival, Prosthesis[Title/Abstract]) OR (Survivals, Prosthesis[Title/Abstract]))
